# Hyaluronan–binding protein 2 (HABP2) gene variation in women with recurrent miscarriage

**DOI:** 10.1186/s12905-018-0618-9

**Published:** 2018-08-24

**Authors:** Frida Husseini-Akram, Sally Haroun, Signe Altmäe, Lottie Skjöldebrand-Sparre, Helena Åkerud, Inger Sundström Poromaa, Britt-Marie Landgren, Anneli Stavreus-Evers

**Affiliations:** 1 Department of Clinical Sciences, Division of Obstetrics and Gynaecology, Danderyds Hospital, Karolinska Institutet, Stockholm, Sweden; 2 Department of Women’s and Children‘s Health, Uppsala University, 751 85 Uppsala, Sweden; 3 Competence Centre on Health Technologies, Tartu, Estonia; 4 Department of Biochemistry and Molecular Biology, Faculty of Sciences, University of Granada, Granada, Spain; 5 Department of Immunology, Genetics and Pathology, Uppsala University, Uppsala, Sweden; 6 Department of Clinical Sciences, Intervention and Technology, Karolinska Institutet, Karolinska University Hospital Huddinge, Stockholm, Sweden

**Keywords:** Genotype, Hyaluronan–binding protein2 (HABP2), Recurrent miscarriage

## Abstract

**Background:**

Idiopathic recurrent miscarriage, defined as three or more consecutive miscarriages, is a distressing early pregnancy complication. Although, the etiology of recurrent miscarriage is still unknown, an aberrant regulation of the endometrial receptivity marker hyaluronan-binding protein 2 (HABP2) has been suggested. The objective of the present study was to investigate the effect of genetic variations of *HABP2* in women with idiopathic recurrent miscarriage compared to fertile women.

**Methods:**

This study was designed as a case-control study. In total, 165 women who had three or more consecutive miscarriages and 289 fertile women were included in the study. Polymorphisms in the *HABP2* gene were analyzed using TaqMan SNP Genotyping Assays. Three polymorphisms in the *HABP2* gene, rs1157916, rs2240879 and rs7080536 (Marburg I) were studied.

**Results:**

Polymorphism in *HABP2* showed no significant difference in women with recurrent miscarriage compared to fertile women, except for rs1157916 minor A allele that was more prevalent among RM patients (*p* = 0.058). Significantly higher live birth rate was observed among women with three to four miscarriages compared to those with more miscarriages (*p* = 0.001).

**Conclusions:**

Variations in the *HABP2* gene did not seem to be involved in the etiology of recurrent miscarriage, while, the number of previous miscarriages had an impact on the live birth rate.

**Electronic supplementary material:**

The online version of this article (10.1186/s12905-018-0618-9) contains supplementary material, which is available to authorized users.

## Background

Recurrent miscarriage (RM), defined as the occurrence of three or more consecutive pregnancy losses, is one of the common causes of subfertility affecting 1–3% of women trying to become pregnant [1, 2]. Many risk factors including uterine abnormalities, endocrine disorders, chromosomal abnormalities [3], autoantibodies, as well as immunological, thrombophilic disorders, genetic disorders, and endometrial factors, have been suggested to be involved in the RM etiology [1, 4, 5], as well as changes in sperm DNA integrity and lifestyle factors (Larsen et al. 2013). However, in 50% of RM cases, none of these factors are found in the couple, and is thus considered to be idiopathic.

One reason for RM might lie in aberrant endometrial receptivity. There is increasing evidence that the endometrium in women with recurrent miscarriage might be more receptive to implantation than in normal fertile controls, and that repeated miscarriage is a result of selection failure in preventing poor quality embryos from implanting, leading to a later recurrent pregnancy loss, hence, repeated miscarriage is a failure to achieve the quality control of bad quality embryos, the so called natural embryo selection [6, 7].

In our previous works, we have consistently identified that *HABP2* transcript plays role in endometrial functions, specifically in endometrial receptivity and embryo implantation process [8–11]. A significant decrease in the expression level of HABP2 at the time of implantation has been observed in patients having miscarriage [12–14]. Further, an earlier study has shown a peak in the distribution of hyaluronan in the stromal compartment during the mid-proliferative and mid-secretory phases of the cycle. This change in hyaluronan deposition and its correlation with the cyclic growth and remodeling of the human endometrium, suggests a major functional role of hyaluronan in this tissue [15]. Having this important role in endometrial functions, HABP2 clearly is a potential candidate for studies of recurrent miscarriage.

The *HABP2* gene, approximately 35 kb in length, is located on chromosome 10q25-q26. It contains 13 exons and 12 introns, with multiple transcription initiation sites that are differentially regulated, and encodes the HABP2 protein, also known as factor VII activating protease (FSAP) [16]. It is an extracellular serine protease, which binds hyaluronic acid; a glycosaminoglycan found abundantly in the female reproductive tract. Hyaluronic acid is a component of the extracellular matrix (ECM), and functions as an angiogenesis promoter [14, 17, 18]. Interestingly it has been shown that women with recurrent pregnancy loss have significantly lower serum HABP2 levels than fertile women [19]. Polymorphisms in the regulatory region of *HABP2* gene have been shown to influence the gene expression levels in the endometrium [8]. Therefore, HABP2 serves as an attractive candidate for studying recurrent miscarriage of unknown origin. In the current study, we aimed to identify the possible associations between genetic variations in *HABP2* in relation to recurrent miscarriage.

## Methods

### Study subjects

All study subjects were recruited from the departments of Obstetrics and Gynecology at Uppsala University Hospital, Karolinska University Hospital, and Danderyd University Hospital, Sweden. The Ethics Boards of Uppsala University and Karolinska Institutet approved the study and all participants gave informed written consent prior to participation.

Eligible cases were identified in the out-patient registers of the participating clinics (with a diagnosis of recurrent miscarriage between 1989 and 2009, depending on the starting point of the out-patient registers at the different centers). Careful review of the medical records was undertaken to assure a correct diagnosis and exclusion of cases with obvious causes for their recurrent miscarriages. Hence, inclusion criterion was three or more verified consecutive miscarriages in the first or second trimester of pregnancy (5–21 completed weeks of gestation), all women were recruited after their third miscarriage. Women with known risk factors for recurrent miscarriages such as Systemic Lupus Erythematosus (SLE), type I diabetes, severe thrombophilia, and major chromosomal aberrations (in either partner) were excluded. Women with type 2 diabetes, any rheumatoid disorder other than SLE, or any autoimmune disease (such as Crohn’s disease or Celiac disease) and coagulopathies other than severe thrombophilia at the time of the initial assessment were kept in the study. All women in this group (*n* = 165) had conceived naturally. The characteristics of the participating women are displayed as mean + SD in (Table 1).

**Table 1:** Clinical characteristics of women with RPL and controls

	RPL *n* = 165	Control *n* = 289	*p*-value
Age	30.1 ± 5.8	30.3 ± 5.9	0.979
Number of miscarriages	4.9 ± 2.4	NA	NA
Number of miscarriages in a row	4.4 ± 2.2	NA	NA
Number of children	1.5 ± 1.2	2.3 ± 0.96	< 0.001
Children before miscarriage	52 (31%)	NA	NA
Children after miscarriage	127 (77%)	NA	NA

Data on age, number of miscarriages, number of miscarriages in a row and number of children is presented as mean ± SD and data on children before and after miscarriage is presented as n (%). Student’s t-test was used for statistical evaluation where applicable. For women with RPL, age was calculated from the age at first miscarriage. *NA* Not Applicable

Control subjects were healthy fertile women (*n* = 289) with no previous history of miscarriage and at least one full term pregnancy. Within the control group 143 were healthy pregnant women that were enrolled in the study in the second trimester of their pregnancy (16–18 weeks of gestation). None of the women included, both cases and controls, had any previous IVF treatment and all included women were of Caucasian ethnicity of European origin. The follow-up time for calculation of pregnancy outcome was two years.

### DNA preparation

Peripheral blood samples were collected in EDTA tubes and stored at − 20 °C until DNA extraction. All samples were extracted after all samples had been collected, and at the same time. Genomic DNA was extracted from whole blood in accordance to the QIAamp® DNA Blood Midi/Maxi Handbook (QIAGEN, Netherlands). Five to ten ml of blood was added to 500 μl of QIAGEN protease, the volume was brought up to 10 ml using PBS. Twelve ml of the buffer AL was added with shaking for at least 1 min to ensure complete lysis, and incubated at 70 °C for 10 min. Then 10 ml of 96% ethanol was added to the samples, and thoroughly mixed in order to ensure efficient DNA binding. The lysate solution was carefully transferred into the QIAamp Maxi column and centrifuged at 3000 rpm for 3 min. The filtrate was discarded and 5 ml of AW1 buffer was added followed by centrifugation at 4000 rpm for 2 min. Then 5 ml of AW2 buffer was added, followed by centrifugation at 4000 rpm for 20 min, to evaporate all traces of AW2 buffer from the column. Prior to elution the QIAamp Maxi column was placed in a clean tube, 600 μl of AE buffer was directly pipetted onto the column’s membrane and incubated at room temperature for 5 min and centrifuged at 4000 rpm for 4 min. This step was repeated twice to obtain a maximum yield of the DNA, and then followed by centrifugation at 4000 rpm for 10 min; aliquots of the DNA samples were prepared and stored at − 20 °C until analyzed.

### Analysis of gene variation

Three polymorphisms in the *HABP2* gene were analyzed: rs1157916 (promoter region), rs2240879 (5”UTR region) and rs7080536 (Marburg I, Gly534Glu in exon 13). Besides the Marburg I polymorphism, which has been shown to influence gene expression, our aim was to study common variation (minor allele frequency > 5%) in the regulatory area of the gene. Selection of SNPs was based on data from literature and from NCBI database. Real-time PCR was performed in accordance to TaqMan® SNP Genotyping Assays manufactures’ instruction kit (Applied Biosystems, USA), using 1 to 20 ng of purified genomic DNA as a template. All samples were analyzed at the same time. The reaction mix was prepared using 2X TaqMan Universal PCR Master Mix 12.5 μl/well, 40X Primer and TaqMan Probe SNP Genotyping Assay diluted 1:4 in TE buffer and 1.25 μl of the solution was added per well, DNase free water 10.25 μl/well, 24 μl of the reaction mix was pipetted into the wells of an optical Micro Amp® 96-well reaction plate purchased from Applied Biosystem, and 1 μl of wet DNA was added, giving a final volume of 25 μl per well. The plate was sealed with a plastic cover, and centrifuged to spin down the contents and eliminate air bubbles. The plate design was performed using Applied Biosystems Step One Software version 2.0, PCR was performed on Step One Plus™ Real-Time PCR System (Applied Biosystems, Foster City, USA). Amplification during 40 cycles with denaturation temperature of 95 °C and annealing temperature of 60 °C was performed. Primers and probes were designed for two of the *HABP2* polymorphisms using the Primer Express® Software Version 3.0 for allelic discrimination assays in accordance to the Applied Biosystems instruction manual, and diluted according to the Custom TaqMan® SNP Genotyping Assays Protocol. Two non-template controls with DNase free water were used to detect possible contamination and two of the tested samples were randomly chosen and duplicated to check the experiment accuracy. The output was plotted in a graph where the outcome of allelic discrimination was evaluated.

### Statistical analysis

For allele frequencies deviations from Hardy-Weinberg equilibrium were investigated. Nominal variables, categorical variables, genotypes, allele frequencies were analyzed using chi-square test. Differences within and between groups were analyzed using one-way ANOVA Test. Missing data was excluded from calculations Data analysis was performed using SPSS Predictive Analytics Software PASW Statistics version 18.0 (SPSS Inc., Chicago, Illinois), a *P* value< 0.05 was considered statistically significant.

## Results

**Fig. 1:**
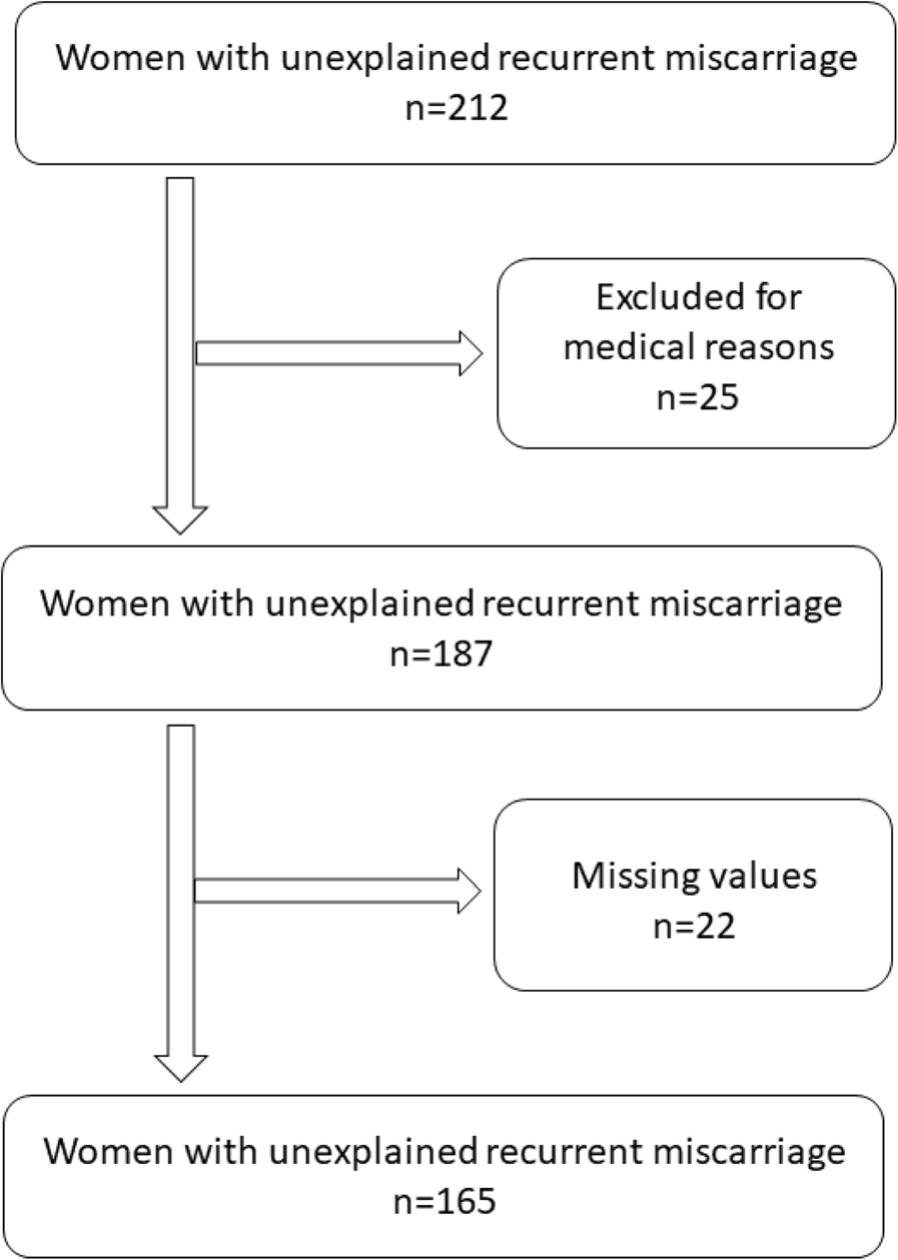
Flow chart of patients included in the study. Medical reasons for drop-out was systemic lupus erythematosus, diabetes type 1, severe thrombophilia, major chromosomal abnormalities, previous infertility treatment, other autoimmune disease or any other severe disease

**Fig. 2:**
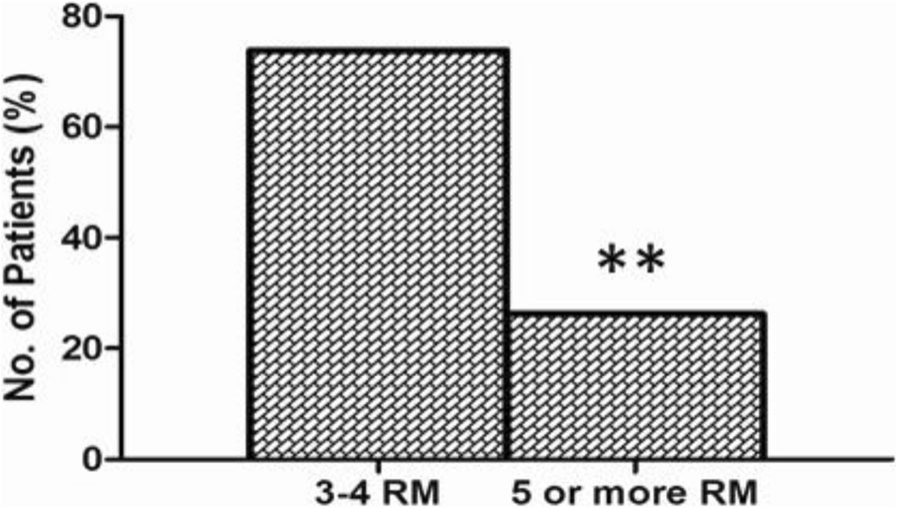
Live birth after recurrent miscarriage (RM). The graph illustrates that women with three to four consecutive miscarriages had a higher live birth rate (73.8%) than women with five or more consecutive miscarriages (*P* value = 0.017)

In this study, 212 women with RPL were recruited. Of these, 165 were included in the study (Fig. 1). There was no difference in age between the two groups while the controls had significantly higher number of children. Pregnancy outcome in women with RM revealed that 77% of the women eventually had a full term pregnancy with at least one child successfully born (Table 1), while women with five or more miscarriages had less chance, 26.2%, of a successful pregnancy outcome compared to 73.8% in the whole group (Fig. 2).

A significant correlation was detected within the RM group between the age at first miscarriage and live births after miscarriage (*P* = 0.001). In women with no children the mean number of repeated miscarriage was 5.19 ± 3.40 compared to women with children, the number was significantly smaller, 4.17 ± 1.76 (*P* = 0.017).

### Gene variation

The genotype and allele frequencies of the *HABP2* polymorphisms in women with recurrent miscarriage and fertile controls are shown in (Table 2). All the studied polymorphisms were in Hardy-Weinberg equilibrium, except for the rs7080536 in patient group. No significant difference was observed between the two groups. However, rs1157916A allele showed borderline significance being more frequent among RM patients (*P =* 0.058). One of the RM patients was homozygous for the Marburg I minor allele (AA). This otherwise healthy patient had a history of five consecutive miscarriages and one live birth before the miscarriages.

**Table 2:** Genotype and allele frequencies of single nucleotide polymorphism in the *HABP2* gene of women with recurrent miscarriage and fertile controls

Allele	Genotype	Frequency (n)	Genotype	Frequency (n)	*p-value*
rs1157916	*n* = 165		*n* = 265		
	GG	37.0% (61)	GG	37.7% (97)	
	GA	45.4% (75)	GA	53.5% (134)	
	AA	17.6% (29)	AA	8.8% (34)	0.348
	*p*(G)	0.597	*p*(G)	0.652	
	*q*(A)	0.403	*q*(A)	0.348	0.058
rs2240879	*n* = 165		*n* = 289		
	AA	46.7% (77)	AA	39.3% (143)	
	AG	43.0% (71)	AG	51.4% (121)	
	GG	10.3% (17)	GG	9.3% (25	0.773
	*p*(A)	0.682	*p*(A)	0.704	
	*q*(G)	0.318	*q*(G)	0.296	0.264
rs70805366	*n* = 162		*n* = 278		
	GG	94.4% (153)	GG	95.3% (265)	
	GA	4.9% (8)	GA	4.7% (13)	
	AA	0.6% (1)	AA	0.0% (0)	0.419
	*p*(G)	0.970	*p*(G)	0.978	
	q(A)	0.030	q(A)	0.022	0.279

Statistics according to Anova, *p* < 0.05 was considered significant difference

## Discussion

The main finding of the study is that the distribution of the different genotypes in *HABP*2 gene was similar in the group with RM and in controls. However, minor allele A the polymorphism in the promoter area, rs1157916, tended to be more frequent among RM patients than in controls. The function of this variant in the regulatory region of the gene is not known, but polymorphism in the noncoding 5’ UTR area can affect promoter usage and thereby can influence protein expression [20]. In fact, among infertile women with unexplained infertility, we detected aberrant endometrial HABP2 expression and this group of women presented less frequently the same minor A allele of rs1157916 [8]. Although the etiology of unexplained infertility and recurrent pregnancy loss is not the same, there are studies showing that both might be related to a defect in endometrial receptivity, where similar processes could be involved [7, 10]. It is known that women with RM easily become pregnant possibly due to a more receptive endometrium. Impaired decidualization makes late implantation possible which negatively affects the quality control of embryos, thus causing early placental failure [2]. Also, suggesting that endometrial stromal cells of women with recurrent miscarriage fail to distinguish between low and high quality embryos [21].

Transcriptome studies have shown a significant differential expression of *HABP2* transcript in the receptive phase endometrium, with a significant down-regulation observed in women with unexplained infertility compared to fertile women [8, 22]. Also a significant decrease in the expression level of HABP2 at the time of implantation has been observed in patients having miscarriage [12–14]. Moreover, a significant up-regulation of the *HABP2* gene expression RNA- sequence was noticed to be endometrial epithelial cell-specific in the receptive phase of the menstrual cycle, and is therefore identified as a putative biomarker of mid-secretory endometrium in four datasets, with a protein validation analysis [11].

Polymorphism in the rs7080536 (Gly534Glu, Marburg I in exon 13, results in a single amino acid substitution of glycine to glutamic acid in the protease domain [23]. Five percent of the Caucasian population are carriers of this SNP, which has been shown to influence gene expression of *HABP2*, and is associated with low proteolytic activity of the protein, resulting in a protease with a weak activation potential of pro-urokinase [24, 25]. In this study one patient was homozygous for the Marburg I minor allele (AA). This otherwise healthy patient had a history of five consecutive miscarriages and one live birth before the miscarriages.

Women with RM in our study had more than a 70% chance of a successful pregnancy. However, the chance of pregnancy decreased after 5 or more miscarriages. This might be due to an age factor or a variation in endometrial receptivity or possibly also that there is a subgroup who will not have a successful pregnancy regardless of the number of attempts. Interestingly, a recently published study demonstrates that women who suffer a miscarriage in their first infertility treatment cycle (in vitro fertilization) have a good chance of a live birth in subsequent cycles when compared to women with no pregnancy establishment (Cameroun, N, 10.1093/humrep/dex293, in press). An important finding in our study, being in line with previous studies, [5, 26] show that there is hope for successful pregnancy outcome for almost 80% of women suffering from recurrent miscarriage.

The limitation of the current study is the relatively small sample size, however, the patient group of RM is hard-to-collect (affecting 1–3% women in their reproductive age). While the fact that this patient group is unique and is very well-defined is the strength of our study.

## Conclusion

Recently, a better understanding of implantation and maternal embryo interactions, as well as progress in the fields of cytogenetics and immunogenetics has provided new insights into the possible underlying causes of repeated miscarriage. HABP2 has important roles in uterine functions and it serves an attractive candidate for RM. Nevertheless, the studied *HABP2* gene variants do not seem to be an important contributing factor in recurrent miscarriage. Further studies, however, on a bigger sample size and also on protein level, including functional studies of gene variants would clarify the role of HABP2 in RM. Further, our study results confirm that the number of previous miscarriages has an impact on the live birth rate in the patient group of RM, giving hope for successful pregnancy outcome for almost 80% of women suffering from recurrent miscarriage.

## Additional file

Additional file 1: SNP raw data for cases and controls is shown. (XLSX 22 kb)

## Abbreviations

ECM: Extracellular matrix
HABP2: Hyaluronan-binding protein 2
HRG: Histidine-rich glycoprotein
KHDC3l: KHDC3-like protein
NLRP: NLR protein
RM: Recurrent miscarriage
SLE: Systemic Lupus Erythematosus
SNP: Single nucleotide polymorphism

## References

[1] Kolte AM (2011). A genome-wide scan in affected sibling pairs with idiopathic recurrent miscarriage suggests genetic linkage. Mol Hum Reprod.

[2] Teklenburg G, et al. The molecular basis of recurrent pregnancy loss: impaired natural embryo selection. Mol Hum Reprod. 2010;16(12):886–95. 10.1093/molehr/gaq079 20847090

[3] Ljunger E, et al. Ultrasonographic findings in spontaneous miscarriage: relation to euploidy and aneuploidy. Fertil Steril. 2011;95(1):221–4. 10.1016/j.fertnstert.2010.06.018 20638056

[4] Li TC, Tuckerman EM, Laird SM (2002). Endometrial factors in recurrent miscarriage. Hum Reprod Update.

[5] Li TC (2002). Recurrent miscarriage: aetiology, management and prognosis. Hum Reprod Update.

[6] Quenby S (2002). Recurrent miscarriage: a defect in nature's quality control?. Hum Reprod.

[7] Teklenburg G (2010). Natural selection of human embryos: decidualizing endometrial stromal cells serve as sensors of embryo quality upon implantation. PLoS One.

[8] Altmae S (2011). Variation in hyaluronan-binding protein 2 (HABP2) promoter region is associated with unexplained female infertility. Reprod Sci.

[9] Altmae S (2012). Research resource: interactome of human embryo implantation: identification of gene expression pathways, regulation, and integrated regulatory networks. Mol Endocrinol.

[10] Altmae S (2010). Endometrial gene expression analysis at the time of embryo implantation in women with unexplained infertility. Mol Hum Reprod.

[11] Altmae S (2017). Meta-signature of human endometrial receptivity: a meta-analysis and validation study of transcriptomic biomarkers. Sci Rep.

[12] Bersinger NA (2008). Gene expression in cultured endometrium from women with different outcomes following IVF. Mol Hum Reprod.

[13] Cordo-Russo R (2009). Murine abortion is associated with enhanced hyaluronan expression and abnormal localization at the fetomaternal interface. Placenta.

[14] Babayan A (2008). Hyaluronan in follicular fluid and embryo implantation following in vitro fertilization and embryo transfer. J Assist Reprod Genet.

[15] Salamonsen LA, Shuster S, Stern R (2001). Distribution of hyaluronan in human endometrium across the menstrual cycle. Implications for implantation and menstruation. Cell Tissue Res.

[16] Sumiya J (1997). Isolation and characterization of the plasma hyaluronan-binding protein (PHBP) gene (HABP2). J Biochem.

[17] Hambiliki F, et al. Hyaluronan-enriched transfer medium in cleavage-stage frozen-thawed embryo transfers increases implantation rate without improvement of delivery rate. Fertil Steril. 2010;94(5):1669–73. 10.1016/j.fertnstert.2009.10.019 19939373

[18] Loutradi KE (2007). Evaluation of a transfer medium containing high concentration of hyaluronan in human in vitro fertilization. Fertil Steril.

[19] Riondino S (2012). Factor seven activating protease activity levels in women with recurrent pregnancy loss. Reprod Sci.

[20] Westberg L (2003). Association between a dinucleotide repeat polymorphism of the estrogen receptor alpha gene and personality traits in women. Mol Psychiatry.

[21] Weimar CH (2012). Endometrial stromal cells of women with recurrent miscarriage fail to discriminate between high- and low-quality human embryos. PLoS One.

[22] Altmae S, et al. Variation in hyaluronan-binding protein 2 (HABP2) promoter region is associated with unexplained female infertility. Reprod Sci. 18(5):485–92. 10.1177/1933719110388849 21098215

[23] Stephan S (2008). Tests for the measurement of factor VII-activating protease (FSAP) activity and antigen levels in citrated plasma, their correlation to PCR testing, and utility for the detection of the Marburg I-polymorphism of FSAP. Clin Chem Lab Med.

[24] Kanse SM (2008). Factor VII-activating protease (FSAP): vascular functions and role in atherosclerosis. Thromb Haemost.

[25] Tag CG (2007). Rapid genotyping of the G534E polymorphism (Marburg I) of the gene encoding the factor VII-activating protease (FSAP) by LightCycler PCR. Clin Biochem.

[26] Li TC (2002). An analysis of the pattern of pregnancy loss in women with recurrent miscarriage. Fertil Steril.

